# Therapeutic Potential of Bipolar Androgen Therapy for Castration-Resistant Prostate Cancer: In Vitro and In Vivo Studies

**DOI:** 10.3390/biomedicines12010181

**Published:** 2024-01-15

**Authors:** Jiwoong Yu, Joung Eun Lim, Wan Song

**Affiliations:** 1 Department of Urology, Samsung Medical Center, Sungkyunkwan University School of Medicine, Seoul 06351, Republic of Korea; 2 Samsung Biomedical Research Institute, Samsung Medical Center, Sungkyunkwan University School of Medicine, Seoul 06351, Republic of Korea

**Keywords:** prostate cancer, castration resistant, bipolar androgen therapy, supraphysiologic, testosterone

## Abstract

Androgen deprivation therapy (ADT) is a primary treatment for advanced prostate cancer (PCa), but resistance often leads to castration-resistant PCa (CRPC). CRPC remains androgen receptor (AR)-dependent, and AR overexpression causes vulnerability to high doses of androgen in CRPC. Bipolar androgen therapy (BAT) refers to the periodic administration of testosterone, resulting in oscillation between supraphysiologic and near-castrate serum testosterone levels. In this study, we evaluated the efficacy of BAT against CRPC in a preclinical setting. To emulate CRPC characteristics, PCa cell lines (LNCaP, VCaP, and 22Rv1) were cultured in phenol red-free RPMI-1640 medium supplemented with 10% dextran-coated charcoal treated FBS (A− cell line). Cell viability, AR, and AR-V7 expression were evaluated using the Cell Counting Kit-8 and Western blotting. In vivo studies involved 12 castrated NOG mice injected with LNCaP/A− cells, treated with testosterone pellets or controls in 2-week cycles. Tumor sizes were measured post a 6-week treatment cycle. Bicalutamide inhibited PCa cell viability but not in the adapted cell lines. Supraphysiologic androgen levels suppressed AR-expressing PCa cell growth in vitro. In vivo, high AR-expressing LNCaP cells proliferated under castrate conditions, while BAT-treated xenografts exhibited significant growth inhibition with low Ki-67 and mitotic indexes and a high cell death index. This study provides preliminary evidence that BAT is effective for the treatment of CRPC through rapid cycling between supraphysiologic and near-castrate serum testosterone levels, inducing an anti-tumor effect.

## 1. Introduction

Prostate cancer (PCa) is the most commonly diagnosed cancer in the United States, accounting for approximately 29% of all cancer diagnoses and 11% of estimated deaths, according to Cancer Statistics 2023 [1]. PCa is an androgen-driven cancer, and androgen deprivation therapy (ADT) is the standard of care for men with advanced and metastatic PCa [2,3,4]. However, although PCa is initially sensitive to ADT, it gradually becomes resistant and ultimately reaches the final stage called castration-resistant PCa (CRPC) [5]. Extensive research efforts have been made to overcome the phase of CRPC, in which PCa proliferates despite low testosterone levels. New strategies introduced in the past decades, such as the combination of ADT and chemotherapy with docetaxel and/or androgen receptor-targeted agents (ARTA) such as enzalutamide or abiraterone acetate, have led to significant survival benefits [6,7,8,9]. However, despite the initial efficacy of ARTA, most patients develop resistance in the short term. More recent treatments, like olaparib, which belong to DNA damage repair-related poly ADP ribose polymerase inhibitors, offer only a modest extension in delaying disease progression [10]. In this context, investigations aimed at identifying therapies for CRPC are actively ongoing. Despite progression to the CRPC stage, PCa cells persist in their reliance upon androgen receptor (AR) signaling to promote proliferation [11]. The most prevalent molecular alteration observed in CRPC involves a 2–4-fold increase in AR expression, which characterizes both the dependence and adaptation of CRPC cells on AR signaling for growth [11,12,13,14]. Conversely, the overexpression of the AR in CRPC creates a therapeutic vulnerability to high-dose androgen therapy. In preclinical models, when confronted with elevated AR expression, the administration of testosterone to attain supraphysiological serum levels paradoxically leads to PCa cell death and tumor regression [15,16,17].

Based on these preclinical findings, clinical trials are currently evaluating the therapeutic potential of a novel treatment strategy for CRPC known as bipolar androgen therapy (BAT). BAT involves the periodic administration of testosterone, which causes fluctuations in serum testosterone levels between supraphysiological and near-castrate concentrations [18,19,20]. A recent systematic review of 10 clinical trials of BAT in CRPC demonstrated the promising efficacy of this strategy. A reduction in prostate-specific antigen (PSA) of >50% was observed in 27% of patients, and the objective response rate was 34%, indicating a significant clinical benefit [21]. However, the mechanisms underlying these responses remain incompletely understood.

In this study, we consolidated preclinical data to evaluate the efficacy of BAT and support its clinical application in patients with CRPC. We validated the therapeutic potential of BAT in PCa cell lines and animal models, and explored the underlying mechanisms.

## 2. Materials and Methods

### 2.1. Chemicals and Reagents

Bicalutamide (≥95% purity) was obtained from Selleck Chemicals (Houston, TX, USA). R1881 (metribolone) was purchased from Sigma-Aldrich (St. Louis, MO, USA). Testosterone pellet was purchased from Belma Technologies (Liège, Belgium). Antibody against AR was purchased from Santa Cruz Biotech. Antibody against AR-V7 was a product of GeneTex (Irvine, CA, USA).

### 2.2. Cell Culture

The human PCa cell lines LNCaP, VCaP, and 22Rv1 were purchased from the American Type Culture Collection (Manassas, VA, USA). LNCaP and 22Rv1 cells were cultured in RPMI-1640 medium (Invitrogen, Waltham, MA, USA), and VCaP cells were cultured in MEM (Invitrogen). Media were supplemented with 10% fetal bovine serum (FBS), penicillin (100 U/mL), and streptomycin (100 mg/mL), and cells were cultured at 37 °C in a humidified atmosphere with 5% CO_2_. Low androgen-adapted PCa cell lines (LNCaP/A−, VCaP/A−, and 22Rv1/A−) generated from the three human prostate cancer cell lines were cultured for 7 days in an androgen depleted environment, which was phenol red-free RPMI-1640 medium (Invitrogen) supplemented with 10% dextran-coated charcoal (DCC)-treated FBS (Invitrogen).

### 2.3. Cell Proliferation Assessment

Cells were plated in 96-well plates at a density of 5000 cells/well and grown overnight. The medium was replaced with a fresh medium containing the indicated compounds, and the cells were incubated for an additional 72 h. Cell viability was determined using the Cell Counting Kit-8 (Dojindo, Kumamoto, Japan) following the manufacturer’s procedures.

### 2.4. Western Blot Analysis: Screening of AR and AR-V7 Protein Expression

PCa cells were cultured in FBS or DCC-FBS medium in 6-well plates. After 24 h, the cells were treated with vehicle or R1881 (1 or 10 nM) for 24 and 48 h. LNCaP and LNCaP/A− cells were treated with 10 nM R1881 or 10 µM bicalutamide for 24 h and then harvested. The cells, whether treated with the indicated drugs or untreated, were lysed in RIPA buffer (Millipore, Billerica, MA, USA) supplemented with a protease inhibitor. The protein concentration was quantified using the Bradford assay (Bio-Rad, Hercules, CA, USA). Aliquots containing 25 µg of total protein were loaded onto 10–15% SDS-PAGE gels and transferred to polyvinylidene difluoride membranes (Millipore, Bedford, MA, USA). The membranes were probed with one of the following primary antibodies: AR (Santa Cruz Biotechnology, Dallas, TX, USA) or AR-V7 (GeneTex, Hsinchu, Taiwan), and β-actin (Cell Signaling Technology, Danvers, MA, USA) at 4 °C overnight. The detection of specific antibody binding involved a 2 h incubation at room temperature with horseradish peroxidase-conjugated anti-rabbit or anti-mouse antibodies. Immunoreactive bands were visualized using the ECL-Plus Kit (Thermo Scientific, Rockford, IL, USA).

### 2.5. In Vivo Studies

Twelve 4-week-old male NOG mice (Koatech, Pyungtaek, Korea) were maintained in compliance with the Guide for the Care and Use of Laboratory Animals prepared by the Institute of Laboratory Animal Resources (National Institutes of Health). All animal procedures (Protocol No. 20211118002) were conducted with the approval of the IACUC (Institutional Animal Care and Use Committee) of the Samsung Medical Center and in alignment with the Animal Experiment Guidelines of the Samsung Biomedical Research Institute, guided by the Institute of Laboratory Animal Resources and certified with Accreditation No. 001003 from AAALAC International.

LNCaP/A− cells (1 × 10^7^ cells/100 µL) were suspended in equal volumes of Matrigel and Hank’s Balanced Salt Solution with Matrigel (BD Biosciences Pharmingen, Franklin Lakes, NJ, USA). The 12 NOG mice, which were castrated, were injected subcutaneously with 200 µL of the cell suspension into the flank region. Upon the tumor volume reaching approximately 100 mm^3^, the mice were randomly divided into two groups, with six mice in each group. One group was treated by implanting testosterone pellets (Belma Technologies, Liège, Belgium) subcutaneously in the abdominal region, while the control group received no treatment.

The testosterone pellets were removed at 2 weeks and then replaced 2 weeks later. Serum was consecutively obtained through the retro-orbital plexus using microhematocrit capillary tubes every two weeks, either before the implantation or removal of the testosterone pellets. After harvest, each tumor specimen was divided into two: one part was fixed in buffered formalin, and the other part was frozen in liquid nitrogen and maintained at −80 °C until further processing. The tumor dimensions were measured using calipers, and the tumor volume was estimated according to the following formula: (longest diameter) × (shortest diameter)^2^/2.

### 2.6. Measurement of PSA and Testosterone Levels

The levels of testosterone in mouse serum were determined by enzyme linked immunosorbent assay according to the technical manual provided with the kit (Abcam Cat. No. ab108666). The PSA serum levels at tumor harvest (42 days) were measured using a PSA ELISA kit (Invitrogen Cat. No. EHKLK3T).

### 2.7. Immunohistochemical Staining

Formalin-fixed, paraffin-embedded serial sections (4 µm) were obtained from the tumor samples. The slides were incubated with primary antibodies against AR (50:1, Santa Cruz Biotechnology) and Ki-67 (100:1, Signaling Technology). The slide sections were then analyzed using an EnVision+ Dual Link Kit (Dako, Glostrup, Denmark) and counterstained with hematoxylin. The cell mitotic or death index was determined by calculating the ratio between the number of Ki-67-positive or TUNEL-positive cells and the total cell count within high-power (×400) fields.

### 2.8. Statistical Analysis

The results are presented as the mean ± standard error of the mean (SE). We utilized the Student’s *t*-test along with the Levene’s equality of variance test to evaluate and compare the means between the control group and the BAT-treated group. Values with *p* < 0.05 were considered statistically significant.

## 3. Results

### 3.1. AR Expression

**Figure 1:**
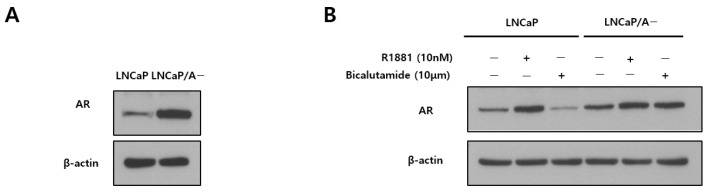
Androgen receptor (AR) levels in LNCaP cells vs. low androgen-adapted LNCaP/A− cells. (**A**) Immunoblot analysis of AR in LNCaP vs. LNCaP/A− cells. (**B**) LNCaP cells and LNCaP/A− cells were treated with R1881 or bicalutamide at the indicated concentrations and analyzed by immunoblotting with the AR antibody.

**Figure 2:**
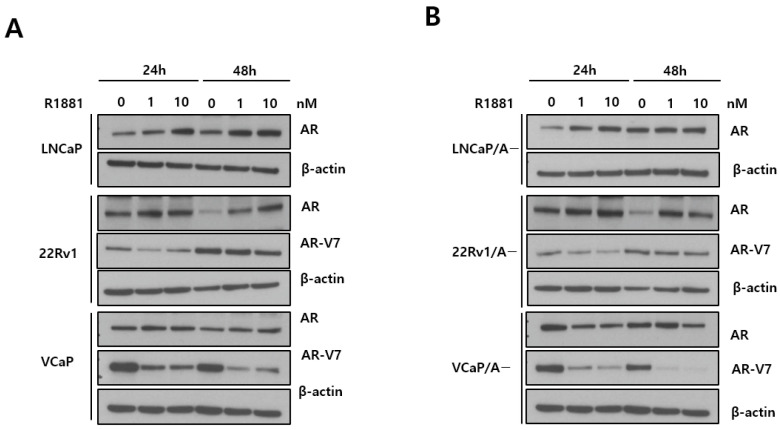
AR and AR-V7 protein expression in human prostate cancer cell lines. (**A**) Immunoblot analysis of AR and AR-V7 in human prostate cancer cell lines treated with the indicated concentrations of R1881 for the indicated times. (**B**) Immunoblot analysis of AR and AR-V7 in low androgen-adapted prostate cancer cell lines.

AR overexpression was detected in LNCaP/A− cells but not in LNCaP cells (Figure 1A). R1881, a synthetic androgen, upregulated AR, and bicalutamide, an antiandrogen, downregulated AR in LNCaP cells, whereas exposure to these drugs had no effect on the LNCaP/A− cells (Figure 1B). The effect of R1881 on upregulating AR expression in the LNCaP, 22Rv1, and VCaP cell lines was dose dependent (Figure 2A), whereas the adapted cell lines (LNCaP/A− and 22Rv1/A−) did not show an obvious increase in AR expression (Figure 2B). In the VCaP/A− cell line, the effect of R1881 on AR expression did not differ significantly between exposure to 1 and 10 nM for 24 h; however, at 48 h, AR expression decreased in response to 10 nM compared with 1 nM R1881 (Figure 2B).

### 3.2. Bicalutamide Resistance and R1881 Dose Effects

**Figure 3:**
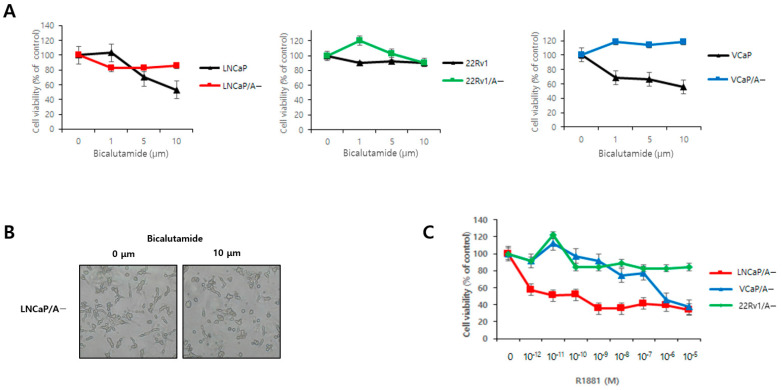
Proliferation rates of prostate cancer cells treated with various compounds. (**A**) Human prostate cancer cell lines were treated with the indicated concentrations of bicalutamide. LNCaP/A−, 22Rv1/A−, and VCaP/A− cells were adapted to grow in a medium with a charcoal-stripped serum. Prostate cancer cell viability was measured using the MTT assay. Error bars represent the mean ± SE. (**B**) LNCaP/A− cells were treated with bicalutamide. (**C**) Dose–response relationship curves of R1881 in LNCaP/A−, 22Rv1/A−, and VCaP/A− cells. Error bars represent the mean ± SE.

Bicalutamide markedly inhibited cell viability in LNCaP and VCaP cell lines, whereas cells adapted to low androgen levels (LNCaP/A− and VCaP/A−) were resistant to bicalutamide-mediated growth inhibition (Figure 3). R1881 decreased LNCaP/A− cell viability in a dose-dependent manner. However, in the VCaP/A−, low concentrations of R1881 increased cell viability, whereas doses exceeding a threshold of approximately 0.1 nM decreased viability. The 22Rv1 and 22Rv1/A− cell lines, which are independent of androgen, exhibited no specific response to both bicalutamide and R1881.

### 3.3. In Vivo Study

**Figure 4:**
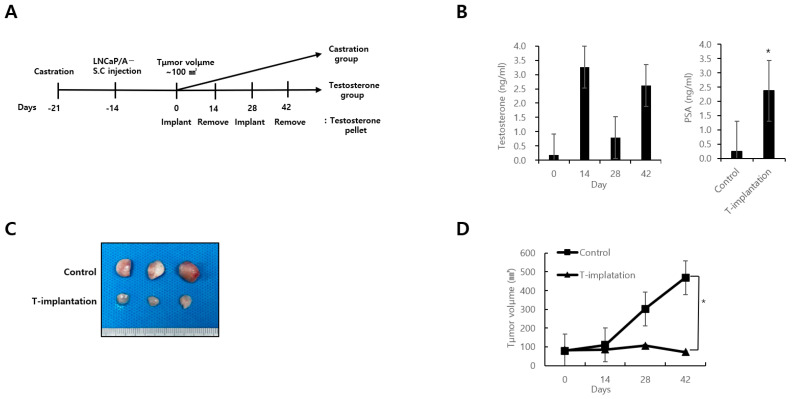
Bipolar androgen therapy inhibits prostate cancer growth in an LNCaP/A− cell xenograft tumor model. (**A**) Schematic of the design of the xenograft model. Castrated male NOG mice (*n* = 12) were inoculated with LNCaP/A− cells. LNCaP/A− cell xenografts were implanted with testosterone pellets for bipolar androgen therapy (*n* = 6). In the castrated animals, implants were placed for 2 weeks, removed for 2 weeks, and then replaced again for 2 weeks before tumor harvest. The other half of castrated mice (*n* = 6) were used as the vehicle controls. (**B**) The total PSA and testosterone levels in mouse serum were determined by enzyme linked immunosorbent assay. * *p* < 0.05. (**C**) Representative photomicrographs of LNCaP/A− cell tumors exposed to bipolar androgen therapy. (**D**) Tumor growth response of LNCaP/A− cells to bipolar androgen therapy. * *p* < 0.05. Supplemental Table S1 contains the detailed mean difference, 95% confidence interval of the difference, and the *p*-value of the comparison.

**Figure 5:**
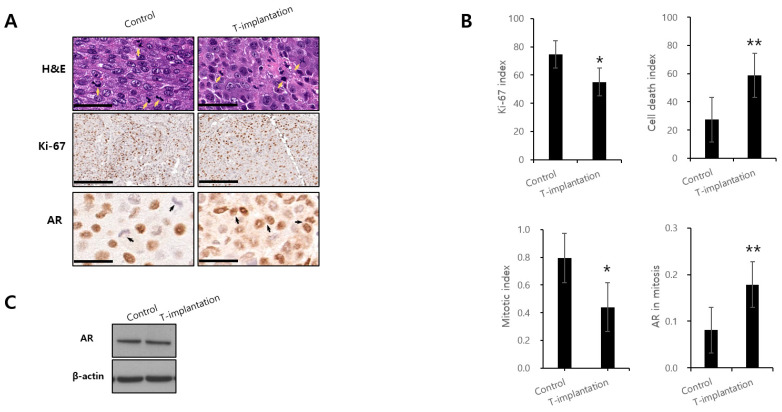
Effects of bipolar androgen therapy in vivo. (**A**) Representative images of hematoxylin and eosin (H&E) (scale bars, 50 μm) and immunohistochemical (IHC) staining for AR (scale bars, 50 μm) and Ki-67 (scale bars, 200 μm). Yellow arrows indicate cancer cell mitoses, while black arrows indicate AR during mitosis. (**B**) Evaluation of the indicated parameters of bipolar androgen therapy in the LNCaP/A− cell xenograft model. * *p* < 0.05, ** *p* < 0.01. (n = 5 fields; mean ± SE). (**C**) Immunoblot analysis of AR in xenograft tumors. Supplemental Table S1 contains the detailed mean difference, 95% confidence interval of the difference, and the *p*-value of the comparison.

In the 12 mice included in the in vivo study, 6 of which received BAT, the serum testosterone levels exhibited oscillations between supraphysiological and castrated levels in two consecutive cycles (Figure 4B). After the final removal of the testosterone pellets, the PSA levels were elevated in the group receiving BAT. In the six mice kept under castrate conditions, tumor xenografts derived from high AR-expressing LNCaP/A− cells demonstrated sustained proliferation, whereas exposure to conditions leading to periodic oscillations between supraphysiological and near-castrate serum testosterone levels resulted in tumor growth inhibition (Figure 4C,D). The BAT-treated xenograft tumors displaying growth inhibition had a lower Ki-67 index and mitotic index and a higher cell death index (Figure 5). AR expression during mitosis was significantly higher in the group that underwent BAT than in the control group.

## 4. Discussion

In this study, in vitro and in vivo experiments confirmed that exposure to synthetic androgen or BAT inhibits the growth of low androgen-adapted cell lines or tumors. BAT resulted in a significant decrease in the mitotic index of tumor cells, along with a notable increase in the cell death index and AR in mitosis compared with the control group. Despite reaching the CRPC stage, the cancer continues to rely on AR signaling for growth as evidenced by the upregulation of AR expression [12,13,14]. In this study, adaptation to low androgen increased AR expression, which was higher in LNCaP/A− than in LNCaP cells (Figure 1A).

Previous research reported that PCa cells with high AR protein expression show pronounced growth inhibition when androgen levels are acutely elevated in the medium [22,23]. Theoretically, supraphysiologic testosterone levels could eliminate CRPC cells with high AR expression, while swift cycling to near-castration testosterone levels would hinder adaptation to high testosterone and eliminate the remaining hormone-sensitive PCa cells expressing low levels of AR. In this study, the viability of VCaP/A− and 22Rv1/A− cells improved under low androgen concentrations, whereas beyond a certain threshold, a decline in viability was observed. Similarly, Song et al. noted that low androgen levels were crucial for the initial growth of PCa cells, while increased androgen concentrations stimulated the proliferation of these cells. Interestingly, a high androgen concentration paradoxically led to a dose-dependent suppression of PCa cell proliferation [24]. These findings suggest that androgens have a biphasic effect on PCa cell growth.

The mechanism underlying the inhibition of PCa growth by high doses of androgen is not well understood. It is known that androgen stabilizes the AR protein and prevents its degradation [25,26]. Consistent with a previous in vitro study [27], our in vivo showed that even during mitosis, supraphysiological testosterone levels prevent AR degradation and AR levels were significantly higher in the group that underwent BAT compared with the control group. The AR functions as a DNA replication licensing factor, akin to other factors in this category [28,29,30]. Licensing factors play a critical role in ensuring that genomic DNA undergoes replication once per cell cycle by assembling on origin of replication (OR) sites during the G1 phase—an essential event for activating replication origins in the S-phase [31]. Similar to other licensing factors, AR undergoes proteasomal degradation during mitosis before entering the subsequent cell cycle [27,29]. However, supraphysiological testosterone conditions prevent the degradation of the AR during mitosis. This inhibition of degradation results in an OR with bound AR, which prevents relicensing and leads to G1 arrest.

On the other hand, androgen stabilizes the AR protein and prevents its degradation while concurrently downregulating AR transcription [26,32]. Hence, the total amount of AR undergoes continuous changes according to the duration of androgen administration. Consistently, in this study, exposure to androgen for 48 h decreased AR levels in the VCaP/A− cell line, in contrast to the results obtained after 24 h of treatment. The abrupt elevation of AR protein levels, whether through direct transcriptional upregulation or ligand-induced stabilization, hinders proper AR degradation during mitosis. This, in turn, prevents the complete relicensing of DNA replication in the subsequent cell cycle. In this regard, the rapid oscillation during BAT is crucial, as it does not allow sufficient time for AR downregulation, thereby affecting DNA replication relicensing and inhibiting the growth of PCa cells.

Several potential mechanisms of cancer cell growth inhibition by supraphysiologic levels of testosterone have been proposed. In one mechanism, the androgen signal promotes the simultaneous recruitment of AR and topoisomerase II b (TOP2b) to locations of TMPRSS2–ERG genomic breakpoints (TEGBs), resulting in recombinant TOP2b-mediated DNA double-strand breaks [33,34]. These breaks lead to gene rearrangement and the activation of apoptosis. Another mechanism suggests that supraphysiological testosterone levels trigger senescence in LNCaP cells, as evidenced by the formation of senescence-associated heterochromatic foci and increased senescence-associated β-galactosidase activity [35,36]. AR and AR splice variants drive CRPC progression, and lysine-specific demethylase 1 (LSD1) demethylates H3K4me1 and H3K4me2 to repress AR gene expression, suggesting that LSD1 inhibition could reduce CRPC cell growth [37,38]. Lastly, castration-resistant LNCaP sublines trigger BAX-mediated apoptosis in response to androgen treatment [39].

Meanwhile, the observation of elevated PSA levels after the removal of the testosterone pellet is attributed to the complexity of evaluating the PSA response in mice undergoing BAT, given that PSA is an androgen-responsive gene. However, in vivo experiments showed a significant reduction in tumor size.

This study has several limitations. Firstly, the experimental design focused on CRPC cell lines that emerged after ADT, and a setting involving additional ARTA use in CRPC was not investigated. Although there are experimental and clinical scenarios in which BAT is attempted directly in CRPC or following ARTA administration, this study used various human PCa cell lines in a CRPC setting and demonstrated effectiveness even before ARTA treatment. This provides a starting point for proposing alternatives in situations in which ARTA use before BAT may not be feasible for various reasons, including economic and regional constraints, in future clinical applications. Secondly, the limited information on how BAT affects the localization of AR, AR signaling pathways, or the tumor microenvironment could pose a constraint. While our research did not specifically delve into localization or the microenvironment, our quantification of AR provided experimental insights into cell growth inhibition. Although this aspect remains unexplored, our findings regarding AR quantification substantiate our understanding of its role in inhibiting cell growth. Finally, using DCC-stripped serum complicates our study by depriving cells not just of androgens but also some nutrients for prostate cell growth. This complicates pinpointing whether bicalutamide-resistant effects solely arise from androgen deprivation or other factors. This study provides only limited information on the implications for understanding resistance mechanisms in CRPC. Also, cell line models of this study may not fully mirror human CRPC due to limited representation of genetic, microenvironmental, temporal, and ethical complexities. Nevertheless, our findings indicate that while bicalutamide did not induce growth in the A− cell line, it did affect cell death at specific concentrations. Interestingly, in the androgen-sensitive LNCaP cells, we noticed a nuanced response in LNCaP/A− cells, showing adaptation to bicalutamide. Although there was a slight decrease at 1 uM, higher concentrations did not significantly impact cell survival. This adaptive behavior led us to infer that these cells might have developed a resistance to bicalutamide, prompting the characterization of CRPC. Translating preliminary evidence supporting efficacy of BAT into clinical practice faces limitations, requiring consideration of side effects and patient management challenges, emphasizing the need for future trials to ensure safety in CRPC patients. These forthcoming studies should investigate cellular-level differences in BAT mechanisms between CRPC with or without ARTA and conduct comprehensive assessments of combined mechanisms. This approach aims to identify the most responsive patient groups and establish optimal cycling terms for BAT implementation.

## 5. Conclusions

This study provides preliminary evidence indicating that supraphysiologic levels of testosterone, as observed in in vitro and in vivo experiments, can inhibit the growth of low androgen-adapted PCa cells by impeding the complete relicensing of DNA replication by undegraded AR. This sheds light on the mechanisms underlying the efficacy of BAT in treating CRPC, and highlights the importance of rapid cycling between supraphysiological and near-castrate serum testosterone levels for inducing a robust antitumor effect.

## Data Availability

The data presented in this study are available on request from the corresponding author.

## Supplementary Materials

The following supporting information can be downloaded at: https://www.mdpi.com/article/10.3390/biomedicines12010181/s1, Supplemental Table S1. Mean differences, 95% confidence intervals, and *p*-values between controls and T-implantation specimens.
