# Supplementary material for: Therapeutic Potential of Bipolar Androgen Therapy for Castration-Resistant Prostate Cancer: In Vitro and In Vivo Studies
Source: Biomedicines. 2024 Jan 15;12(1):181. doi: 10.3390/biomedicines12010181 (PMC10813541; doi:10.3390/biomedicines12010181)
Supplement: Supplementary file 1 [file biomedicines-12-00181-s001.zip › biomedicines-2821681-supplementary.pdf]

Supplemental Table S1. Mean differences, 95% confidence intervals, and p-values between controls and t-implantation specimens

		**Control – T-implantation**		
**Figure**	**Variable**	**Mean difference**	**95% CI of the difference**	**P**
Figure 4B	PSA	-2.12	(-2.87) – (-1.37)	0.037
Figure 4D	Tumor volume	396.56	37.02 – 756.11	0.034
Figure 5B	Ki-67	19.56	1.33 – 37.80	0.039
Figure 5B	Cell death index	-31.44	(-39.63) – (-23.24)	<0.001
Figure 5B	Mitotic index	0.36	0.04 – 0.67	0.035
Figure 5B	AR in mitosis	-0.10	(-0.16) – (-0.04)	0.005
